# A Component Decomposition Model for 3D Laser Scanning Pavement Data Based on High-Pass Filtering and Sparse Analysis

**DOI:** 10.3390/s18072294

**Published:** 2018-07-15

**Authors:** Rong Gui, Xin Xu, Dejin Zhang, Hong Lin, Fangling Pu, Li He, Min Cao

**Affiliations:** 1 School of Electronic Information, Wuhan University, Wuhan 430072, China; ronggui2013@whu.edu.cn (R.G.); flpu@whu.edu.cn (F.P.); 2 Wuhan Wuda Zoyon Science and Technology Co. Ltd., Wuhan 430223, China; linhong0607@163.com (H.L.); 13307100949@189.cn (M.C.); 3 School of Electrical and Electronic Engineering, Hubei University of Technology, Wuhan 430072, China; djzhang@whu.edu.cn (D.Z.); heli.edu@hotmail.com (L.H.)

**Keywords:** 3D laser scanning, components decomposition, pavement distresses and performance indicators, high-pass filtering, total variation de-noising

## Abstract

High-precision 3D laser scanning pavement data contains rich pavement scene information and certain components associations. Moreover, for pavement maintenance and management, there is an urgent need to develop automatic methods that can extract comprehensive information about different pavement indicators simultaneously. By analyzing the frequency and sparse characteristics of pavement distresses and performance indicators—including the cracks, road markings, rutting, potholes, textures—this paper proposes 3D pavement components decomposition model (3D-PCDM) which decomposes the 3D pavement profiles into sparse components ***x***, low-frequency components ***f***, and vibration components ***t***. Designed high-pass filter was first employed to separate ***f***, then, ***x*** and ***t*** are separated by total variation de-noising which based on sparse characteristics. Decomposed ***x*** can be used to characterize the location and depth information of sparse and sparse derived signals such as cracks, road marks, grooves, and potholes in profiles. Decomposed ***f*** can be used to determine the slow deformation of pavement. While decomposed ***t*** reflects the fluctuation of the pavement material particles. Experiments were conducted using actual pavement 3D data, the decomposed components can obtain by 3D-PCDM. The effectiveness and accuracy of the ***x*** are verified by actual cracks and road markings, the accuracy of extracted sparse components is over 92.75%.

## 1. Introduction

With the development of measurement sensors, various technological solutions have been applied to automatically detect and report pavement distress and performance indicators information for government agencies in order to accelerate pavement maintenance and management tasks. Typical asphalt pavement distresses—e.g., cracks [1,2,3], potholes [4,5], rutting [5,6]—often cause significant safety and economic problems [1,7,8,9], while some pavement performance indicators including road markings [10], textures [11,12], and grooves [13,14] have great significance for traffic safety. Moreover, pavement distress and performance indicators are important foundations of intelligent transport systems [14,15,16]. In the past two decades, a number of 2D imaging based systems and associated algorithms for pavement surface measurement have been developed for collecting in situ data to evaluate pavement conditions [17,18,19,20]. However, theses traditional 2D image analysis-based pavement defect distress methods often suffer from their inability to discriminate dark areas not caused by pavement defect such as shadows, poor illumination, etc. [2,3,21,22,23,24]. Moreover, the 2D methods cannot detect some defects due to the lack of depth information [25]. Recently, 3D laser scanning pavement surface data acquisition system that can collect high-precision 3D continuous pavement profiles for constructing pavement surfaces has become available. Compared with the traditional 2D visual based methods and manual survey methods, the 3D pavement condition survey methods have greater advantages in terms of accuracy and comprehensiveness [5,21,22,23,24,25]. 

3D laser scanning systems have proven their ability to obtain the depth information and are less vulnerable to lighting conditions [26,27], 3D laser technology has become the dominant approach to automated pavement data collection in recent years [4,23,24]. A significant amount of high-precision 3D pavement data can be acquired and has been widely employed for pavement distress and performance indicators detection—e.g., cracks [1,3,23,25], potholes [4,5], rutting [4], and texture [28]. In general, most studies published in recent years about pavement condition survey from 3D laser scanning pavement data mainly focused on only one kind of distress or performance indicator of pavement [24]. However, it is urgently needed to develop methods that can extract different types of information about pavement distress and performance indicators simultaneously from pavement data for decision marking [24,29], performance trend evaluation in pavement maintenance and management [29,30]. 

On the other hand, according to the related reports [3,4,25,26,31,32], the depth resolution *R_z* and the transverse resolution *R_x* of the 3D laser scanning measurement sensors can reach 0.5 mm and 1 mm. These imply that there is the rich pavement scene information in such high-precision 3D data. Laurent et al. [1] adopted an auto-synchronized laser scanning system to detect road rutting and cracking. A modification of the dynamic optimization algorithm [3] was implemented to detect cracks on 3D laser scanning pavement surfaces. Hybrid procedures of matched filtering, tensor voting and minimum spanning tree were also developed for crack detection using the 3D pavement data [33]. Typical 3D pavement pothole detection methods include watershed method [4] and Kalman filter [34]. Li et al. [5] have presented a real-time 3D laser scanning system for pavement rutting, shoving detection. From the above existing literatures, it can be concluded that the high precision 3D laser scanning pavement data (*R_z* < 0.5 mm, *R_x* = 1 mm) already has the ability to identify a variety of diseases and pavement indicators. 

However, as the technology is protected, few studies describe algorithms in detail about the laser profile characteristic in terms of profile shape, crack shape, number of cracks, and signal-to-noise ratio [35]. Limited studies have been reported about analysis of compositional characteristics of 3D pavement data, and the correlation between 3D pavement distress and performance indicators have rarely been studied. A 3D shadow modeling was proposed for detection of various descended patterns (i.e., cracks, joints, grooves, and potholes) on 3D pavement surface [24]; however, this method required substantial manual assistance to obtain consistent results, and it was sensitive to local noises [23]. Moreover, the 3D shadow modeling that applied local gradient and local dip made it hard to detect the defects of slow deformation in pavement. It is assumed that the pavement profile signal can be decomposed into a number of components, namely: crack, a main signal, a bump, a rut, a pothole, and a noise [35]. In [36] the main profile is reconstructed very well, however computations are time consuming and its performance has to be improved. Therefore, it is necessary to study components information in 3D pavement data, so as to fully utilize the 3D pavement data and provide a good foundation for subsequent applications to various types of pavement distresses or performance indicators. 

The main challenge in components information analysis from high resolution 3D pavement data comes from the various complex environments [23] and the diversity of components, including pavement distresses and performance indicators. By analyzing the frequency characteristics and sparse characteristics of pavement distresses and performance indicators—including cracks, road markings, rutting, potholes, and textures—a 3D pavement components decomposition model (3D-PCDM) is proposed to decompose the 3D laser scanning pavement profiles into sparse component ***x***, low-frequency component ***f***, and vibration component ***t***. The basic assumption is that the cracks, road markings, and potholes in profiles possess high frequency and sparse (or sparse derived) characteristics, the slow deformation of pavement—e.g., rutting and main profiles possess low frequency characteristics, while the textures in profiles have relative uniform vibration characteristics. Based on these characteristics, components with different properties can be separated from pavement profiles. In this framework, two main problems must be considered: (i) how to define the frequency properties and sparse properties of different components; (ii) how to separate different components adaptively from different profiles, and to extract different types of pavement distress and performance indicators information simultaneously.

Aiming to make full use of rich scene information in high-precision 3D laser scanning pavement data and to obtain different pavement distresses and performance indicators information simultaneously, the proposed 3D-PCDM has the following main steps: firstly, the frequency characteristics and sparse characteristics of pavement distresses and performance indicators are analyzed from the perspective of 3D pavement profiles. Secondly, a designed high-pass filter [37] was first employed to separate the low-frequency component ***f*** from 3D pavement profile data. Thirdly, the sparse component ***x*** and the vibration component ***t*** are separated by total variation de-noising [38] which based on sparse characteristics. Finally, some specific pavement distresses and performance indicators are used to verify the validity of the decomposed components—including the cracks—road markings are used to verify the validity of sparse components, and the effectiveness of the vibration components is verified by pavement texture. The proposed method has the following main novelties and advantages:

- The 3D-PCDM method decomposes the 3D laser scanning pavement profiles into sparse components, low-frequency components, and vibration components by employing the frequency characteristics and sparse characteristics of pavement distresses and performance indicators.
- 3D-PCDM provides a method basis for obtaining different types of information about pavement distresses and performance indicators simultaneously, the decomposed components can all be related to typical real interested pavement defects and performance indicators.

The remainder of the paper is structured as follows: in Section 2, we review the 3D laser scanning pavement data acquisition and introduce the typical components characteristic analysis and total variation de-noising (sparse analysis). In Section 3, we present the workflow and the implementation of the proposed method in detail. In Section 4, experimental tests were conducted using actual pavement 3D data, the decomposed components can obtain by 3D-PCDM. The effectiveness and accuracy of the sparse components are verified by actual cracks and road markings, and the effectiveness of the vibration components is verified by pavement texture, moreover, the capabilities and limitations are discussed. Finally, the conclusions are presented in Section 5.

## 2. 3D Laser Scanning Pavement Data Acquisition and the Components Modeling

In this section, we first review the 3D laser scanning pavement data acquisition. Then, the typical components characteristic analysis is introduced, and we briefly state the possibilities and suitability of employing the total variation de-noising (sparse analysis) to 3D pavement components analysis.

### 2.1. 3D Laser Scanning Pavement Data Acquisition

Based on the laser triangulation principle [2,39], some 3D line laser pavement distress detection systems have been developed in recent years. Here, only the most relevant systems are mentioned. Notable systems, such as the laser crack measurement system (LCMS/LCMS2) [3,31,40,41], have been widely used for automated crack detection on a variety of pavement surfaces [31]. The *PaveVison3D* system mounted in a digital highway data vehicle (DHDV) has been widely used in crack detection related applications [23,24,33]. Another system is Hakeye2000 rapid detection system [42]. Moreover, the laser scanning profiler (LSP) [25,43] has been applied widely in China. In order to obtain high quality pavement data, the aforementioned system sensors are installed in the vehicle for data collection. During data collection, the system can work with speed up about 100 km/h. These systems usually work with resolution that allows the detection of cracks wider than 1 mm. The differences among them are mainly the width of the scanned area, depth, and transverse resolution, as well as the specific distress identification algorithm applied.

**Figure 1:**
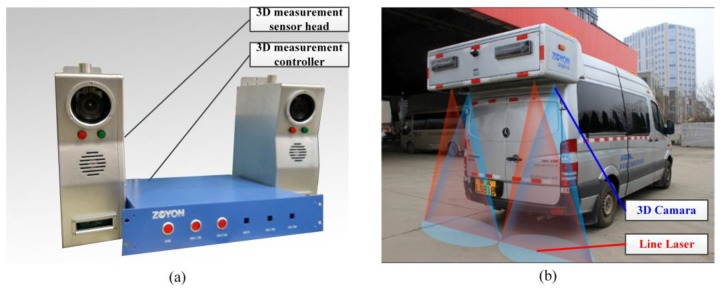
The applied 3D pavement data collection system and the installed version; (**a**) the collection system, (**b**) the system installed version.

In this paper, the applied 3D pavement data collection system is the laser scanning profiler (LSP, ZOYON, Wuhan, China) [25,43], as illustrated in Figure 1. The 3D line laser pavement data collection system is used to measure the transverse pavement elevation profiles consecutively, pre-processing and storing the profile data. The collection system includes the sensor head and the controller. The sensor head obtains the 3D profile data of the measured pavement, while the controller is responsible for controlling the sensor, receiving and pre-processing the profile data, and synchronously uploading the pre-processed data to the host computer. In the dynamic measurement environment, the 3D sensor head is performed by combining the 3D camera and the line laser, in which the line laser shoot vertically onto the measured pavement, and the 3D camera, which is often installed with a certain angle to the line laser, e.g., 6–8°, extracts the laser beam and inference the elevation values with embedded algorithms. After transforming the image coordinates to the object coordinates, that is the calibration, the measured object surface elevation data, i.e., the profiles, are obtained.

In practical application, the 3D pavement data collection system is installed on the vehicle, as shown in the Figure 1b. Typically, this system is required to collect pavement data with a width larger than 3.75 m, and a resolution of 1 mm in the transverse direction (sampling interval is 1 mm in profiles). In the practical working environment, there are two 2K resolution cameras are installed along two sides of vehicle, which can combine to capture the scanned area with a width about 4 m. The speed of pavement inspection is required to meet the normal driving speed range of 0–120 km/h, under a resolution of 1mm along the transverse direction, a depth accuracy of 0.2 mm, a depth measurement range over 0.2 m, and the sampling interval between 1–5 mm along driving direction.

### 2.2. Component Characteristic Analysis in 3D Pavement Data

**Figure 2:**
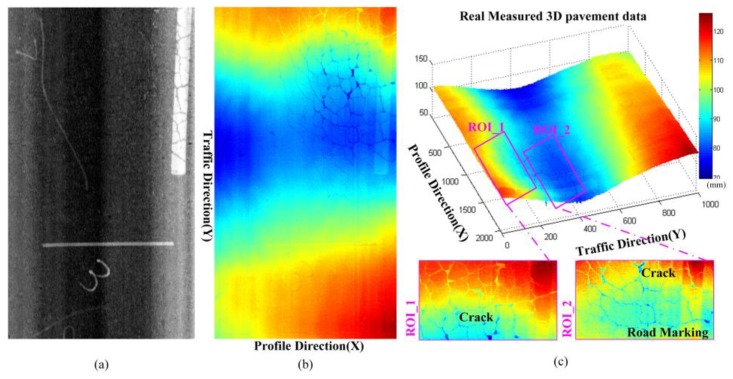
The 2D pavement image and corresponding 3D laser scanning data (with crack, road marking and deformation), (**a**) 2D pavement image; (**b**) real measured 3D laser scanning data; (**c**) 3D laser scanning data and some local ROI illustrations.

**Figure 3:**
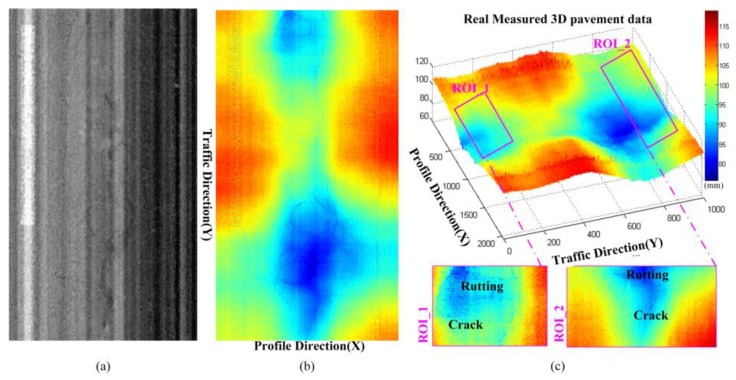
The 2D pavement image and corresponding 3D laser scanning data (with cracks, road markings, and rutting), (**a**) 2D pavement image; (**b**) real measured 3D laser scanning data; (**c**) 3D laser scanning data and some local ROI illustrations.

**Figure 4:**
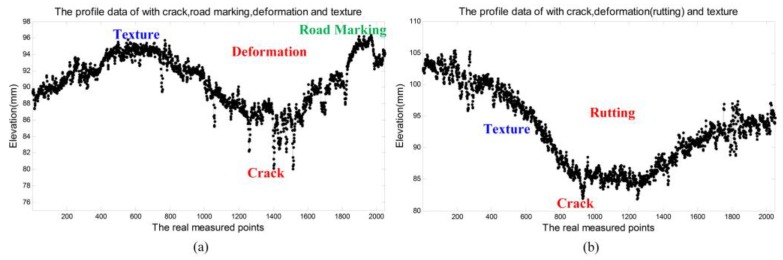
The pavement profile data with different information, (**a**) the profile with cracks, road markings, deformation, and texture; and (**b**) profile with cracks, rutting, and texture.

Figure 2 and Figure 3 illustrate some typical 2D pavement images and corresponding real measured 3D laser scanning data. From the real measured pavement data, it is obvious that the information in 2D pavement images is hard to reflect the real situation and details of the pavement surface due to the lack of depth information. While there are rich and complex pavement information contained in the high-precision 3D laser scanning pavement data, typical information in the selected ROIs including the macro-texture, road markings, cracks, rutting, etc. Moreover, due to the uneven surface, the vehicle vibrates up and down [3,21], there are inevitably included cross-slope information on real measured 3D laser scanning pavement data. The 3D laser scanning data is composed of the continuous measured profile data that reflect the relative elevation information of the pavement cross section [1,2,5,25,35], (the measured profile data is the elevation in profile direction, that perpendicular to the traffic direction of aforementioned vehicle system). Figure 4 shows some profile data from the 3D data in Figure 2 and Figure 3. It can be seen that the elevation fluctuation of pavement texture, the sharp decreases of cracks, slow-varying and wide deformation caused by pavement rutting, the step-shaped elevation characteristics of the road marking, and some cross-slope information.

Because of the high accuracy, high frequency, and high dynamic characteristics of the laser scan 3D pavement system and the various complex pavement environments, the acquired 3D pavement data always contains rich pavement scene information. Typical pavement scene information includes: pavement standard contour information, pavement distresses information, texture (structure depth) information, road markings, and patching information, among which distresses are mainly cracks, potholes, rutting, etc. Moreover, there are certain mutual influence relationships among the various typical pavement scene information. For example, the extraction of 3D pavement cracks is often related to the pavement texture [23,24,25], obtaining the texture information of different pavements can further improve the accuracy of crack extraction. Therefore, when using 3D pavement data to analyze a certain pavement index, it is inevitably needed to obtain information from other related indicators to provide the relevant prior information, in order to extract the certain index more accurately and improve the universality of the method.

**Figure 5:**
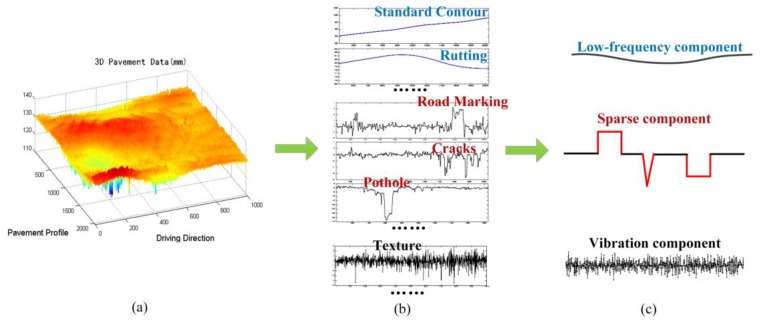
3D pavement data and some typical components, (**a**) 3D pavement data; (**b**) typical pavement distresses and performance indicators from a perspective of ideal profile; and (**c**) some typical components, low-frequency components, sparse components, and vibration components.

Under normal conditions, the slope and curvature of the 3D pavement profiles are small. The frequency information of the standard contour of the pavement can be obtained according to the relevant standards and data resolution information. From the view of signal frequency, the standard profile of the normal pavement profile and the large area with slowly changing, such as the rutting, are low frequency signals. Moreover, the pavement texture and pavement cracks, road markings, and the edge of the potholes, and so on, from its elevation characteristics and its distribution characteristics, this kind of signals have sharply changing and a certain high frequency variation characteristic. On the other hand, because the roughness of the pavement texture has a certain standard type relative to the particle size of the pavement material, and the texture in whole profile is evenly distributed, it can be considered that the pavement texture information ***t*** has more uniform vibration characteristics. Pavement cracks, road markings, and the edges of the potholes usually occupy a relatively small area in elevation profiles; and the cracks, the edge of the markings, the edge of the potholes can be regarded as the sparse signal ***x***. The above analysis of typical pavement information is shown in Figure 5.

According to the characteristics of signal frequency and sparsity, it is assumed that the pavement profiles can be divided into three main components: low frequency information components, sparse components, and vibration components. The low-frequency information includes the pavement standard contour, and slow deformation information. The sparse components include the cracks, the road markings, and the sharply changing pothole edges information. The vibration components are mainly the texture information caused by the road surface material particles. The frequency information of the low frequency components can be obtained according to the relevant standards and data resolution information, the sparse characteristic can be performed by some typical statistical methods, further instructions will be discussed in Section 3. Based on the above assumptions about frequency and sparse characteristics of different components in 3D pavement profile data, this paper proposes a 3D laser scanning pavement data component analysis method based on frequency analysis and total variation de-noising model. The proposed 3D pavement component decomposition model, abbreviated as 3D-PCDM.

### 2.3. Total Variation De-Noising (Sparse Analysis)

The total variation (TV) model was introduced as a regularizing criterion for signal de-noising by Rudin, Osher, and Fatemi [38,44], and has been shown to be quite efficient for smoothing signals while preserving contours. It is based on the principle that signals with excessive and possibly spurious detail have high total variation, that is, the integral of the absolute gradient of the signal is high. According to this principle, reducing the total variation of signal subject to it being a close match to the original signal, removes unwanted detail whilst preserving important details such as edges. 

For a 1D digital signal series $x=\{x_{1},x_{2},\cdots ,x_{n}\}$, we can, for example, define total variation as,

(1) $$V(x)=\sum_{i=1}^{n-1}\left|x_{i+1}-x_{i}\right|$$.

Given an input digital noisy signal ***h***, the goal of total variation de-noising is to find an approximation, call it ***x***, which has smaller total variation than ***h***, but is ‘close’ to ***h***. One measure of closeness is the sum of square errors,

(2) $$E\left(h,x\right)=\frac{1}{2}\underset{n}{\sum }{\left(h_{n}-x_{n}\right)}^{2}$$.

So the total variation de-noising problem amounts to minimizing the following discrete functional over the signal ***x***,

(3) J=E(h,x)+λV(x).

By differentiating this functional with respect to ***x***, we can derive a corresponding Euler–Lagrange equation, which can be numerically integrated with the original signal ***h*** as initial condition. This was the original approach [38]. Alternatively, since this is a convex functional, techniques from convex optimization can be used to minimize it and find the solution ***x*** [45]. The regularization parameter λ adjustable in the de-noising process, further discussion on how to choose λ will be provided in Section 3.3.

## 3. Proposed 3D-PCDM

**Figure 6:**
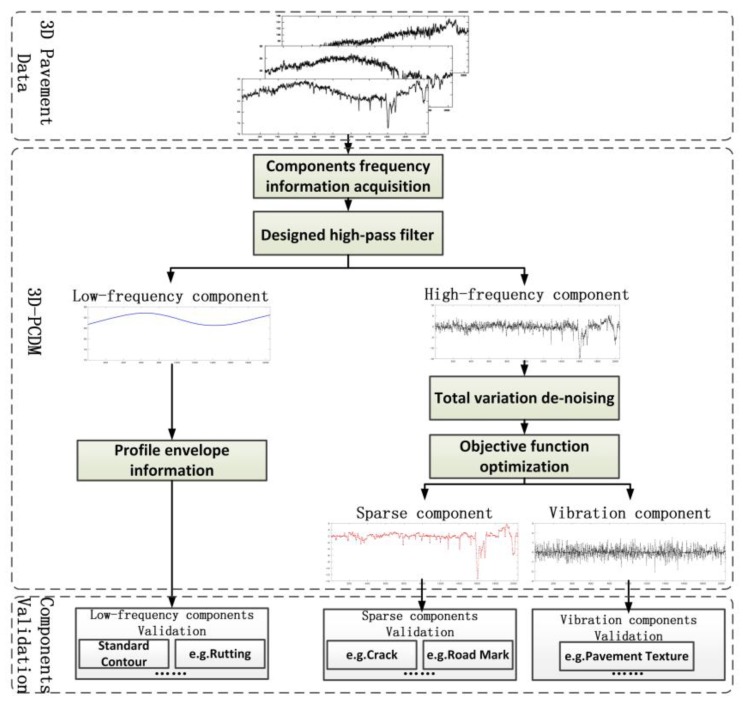
Flowchart of the proposed 3D-PCDM.

The applied workflow (Figure 6) of 3D pavement components decomposition model (3D-PCDM) is organized as follows: After characteristics analysis of pavement distresses and performance indicators, the frequency information that distinguishes the low frequency components and high frequency components can be acquired by relevant standards and data resolution information. Correspondingly, a designed high-pass filter was first employed to separate low-frequency component ***f***. Then, based on sparse characteristics, sparse component ***x*** and the vibration component ***t*** are separated by total variation de-noising and corresponding objective function optimization. The effectiveness and accuracy of the sparse components are verified by actual cracks and road markings, and the effectiveness of the vibration components is verified by pavement texture. While for the low-frequency component, the profile envelop information [43] can be introduced to verify the effectiveness.

In proposed 3D-PCDM, we use ***y*** to represent every profile data, its length is *N*. Every profile data ***y*** can be decomposed into sparse component ***x***, low-frequency component ***f*** and vibration component ***t***, as

(4) y=f+x+t.

In our methodology, we assume that the pavement profile vibration component ***t*** satisfies Gaussian white noise characteristics. More details about the model are as follows.

### 3.1. Component Frequency Information Acquisition

For the acquired profile data ***y***, the fluctuation characteristics are related not only to the physical properties of the components themselves, but also related to the profile data resolution (sampling interval). Since the resolution of pavement 3D data used in this paper is known (profile data length *N* = 2048, transverse resolution *R_x* = 1 mm, elevation resolution *R_z* = 0.2 mm), this part mainly analyzes the fluctuation characteristics of typical components based on the profile data with fixed resolution. The purpose of fluctuation characteristic analysis is to obtain cut-off frequency *fc* between low frequency components and high frequency components in 3D pavement profile data.

Generally, given the factors such as pavement drainage, there is a certain degree of curvature of the asphalt pavement. On the other hand, asphalt pavements often have rutting that change slowly but have a large spatial range. In profile data, rutting is typical low frequency component. Typically, the rutting with depths greater than 10 mm can affect vehicle safety and thus need to be detected. In addition, the width *W_r* of one-sided rutting generally is 0.5–1 m.

(5) T=W_r/R_x

(6) T_m=min(T)

(7) fc=1/T_m

From above analysis, one-sided rutting width is about 0.5–1 m, the profile transverse resolution *R_x* = 1 mm, it is considered that the minimum period *T_m* is 500. Therefore, the cut-off frequency *fc* = 1/500 = 0.002 for the low-frequency components allowed in the profiles. In addition, for the profile data with this resolution, the fluctuation degree of normal pavement should be less than that of rutting. Therefore, it is considered that the difference between the low frequency components and the high frequency components (the sum of the sparse components and the vibration components) of the 3D pavement profile under the resolution is 0.002. Further experimental verification will prove that the way we choose the *fc* abovementioned is reasonable.

### 3.2. Low Frequency Component Separation

A discrete-time filter is described by the following difference equation,

(8) $$\sum_{k}a(k)h(n-k)=\sum_{k}b(k)y(n-k)$$

where y(n) and h(n) are the input and output signals respectively. The frequency response of the discrete-time filter is $H(e^{jw})=B(e^{jw})/A(e^{jw})$, where B(z) and A(z) are the Z-transforms of b(k) and a(k), respectively. To implement the difference for finite-length signals, we write,

(9) Ah=By.

The high-pass filter, ***H***, is taken to be a zero-phase recursive discrete-time filter that we write as $H=A^{-1}B$, where ***A*** and ***B*** are banded Toeplitz matrices, as described in Sec. VI of [46,47]. We further suppose that ***B*** admits the factorization

(10) $$B=B_{1}D$$

where *B_1_* is banded and *D* is the first-order difference matrix

(11) $$D_{(N-1)*N}=\left[\begin{array}{llll} -1 & 1 & & \\ & -1 & 1 & \\ & \;\ddots & \;\ddots & \\ & & -1 & 1 \end{array}\right]$$.

The fact that ***A*** and ***B*** are banded is important for the computational efficiency of the algorithm to be developed. We also assume that ***A***, ***B_1_***, and ***D*** commute.

(12) $$H(z)=\frac{{\left(-z+2-z^{-1}\right)}^{d}}{{\left(-z+2-z^{-1}\right)}^{d}+\alpha {\left(z+2-z^{-1}\right)}^{d}}$$

where α > 0 is related to the cut-off frequency *fc.* This is a zero-phase Butterworth filter of the order 2*d* [37,47,48].

In this work, we set the filter order 2*d* = 2, *f_c_* = 0.002, and the data length *N* = 2048. After the processing of above-described filter ***H***, low-frequency information ***f*** and high-frequency information ***h*** contained in the profile data ***y*** can be acquired.

### 3.3. Sparse Component Separation Based on Total Variation De-Noising

As is well known, the $l_{1}$ norm is a convex proxy for sparsity, so it is practical to formulate sparse-derivative de-noising as the problem of minimizing the $l_{1}$ norm of the derivative of *x* subject to a data fidelity constraint [38]. For discrete-time data, the simplest approximation of the derivative is the first-order difference; hence, the total variation in equation (1) can be considered as the minimization of ${\left\|Dx\right\|}_{1}$, that is,

(13) $$V(x)={\left\|Dx\right\|}_{1}$$.

As mentioned in Section 2.3, given noisy data ***h***, the output of the TV filter is defined as the solution ***x*** to the minimization problem in Equation (3). Combining Equation (3) with Equation (13), we have following optimization equations,

(14) $$J\;=\;min\{\frac{1}{2}{\left\|h-x\right\|}_{2}^{2}+\lambda {\left\|Dx\right\|}_{1}\}$$.

There is no explicit solution to the above equation, but a direct and very fast algorithm to compute the exact solution has been developed [49]. The fast non-iterative algorithm is proposed for de-noising or smoothing one-dimensional discrete signals, by solving the total variation regularized least-squares problem or the related fused lasso problem. A C code implementation is available on the web page of the author [49]. We employed this algorithm to obtain the sparse component ***x*** from the high-frequency information ***h***. The parameter λ, we set its value to 1.25. Since the texture of the asphalt pavement is generally 1–2 mm, this value of λ can be adjusted according to actual situation.

After obtaining the low-frequency information ***f*** and sparse component ***x***, the vibration component ***t = y – f – x***. Thus, the profile data ***y*** can be decomposed into sparse component ***x***, low-frequency component ***f*** and vibration component ***t***. In the following section, we will adopt these three components and verify their effectiveness and accuracy.

### 3.4. Validation Method of Components Effectiveness

In our method assumptions, we consider the obtained components are related to the actual pavement distresses and performance indicators with a certain attribute. For examples, the low-frequency information related to the pavement standard contour, and the slow deformation information. The sparse components include the cracks, the road markings, and the sharply changing pothole edges information. While the vibration components are mainly the texture information caused by the road surface material particles. After continuous profile 3D-PCDM processing and corresponding splicing of the same type of components, thus, the corresponding 3D depth maps of different components can be obtained. Since the characteristics of the decomposed components are relatively simple, it is very beneficial to extract the indicators that possess the same characteristics in corresponding components without being affected by other components. The decomposed components can be reliable foundation for the extraction and analysis of various pavement distresses and performance indicators.

**Figure 7:**
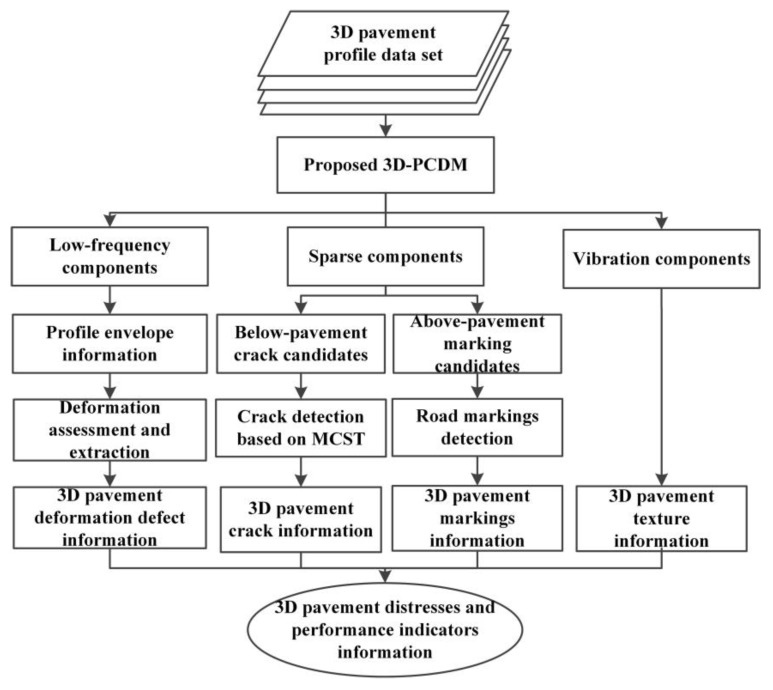
Validation methods of decomposition component effectiveness.

In order to verify the validity of the 3D-PCDM model, we decompose the real measured 3D pavement data, then the corresponding components are employed to extract and analyze the pavement distresses and performance indicators. Thus, the validity of the decomposition components is verified by the accuracy of the corresponding indicators. Figure 7 illustrated the validation methods of decomposition components effectiveness. For the sparse components ***x***, there are mainly two kinds of sparse components in profiles, the below-pavement sparse components include the cracks candidates and the sharply changing pothole edges information, while the above-pavement sparse components include the road marking candidates and some foreign objects information. Then, for the below-pavement sparse components, specifically, the points below the pavement 2 mm are chosen as the crack candidates. Moreover, our previous work minimum cost spanning tree (MCST) algorithm [17,25] was further employed to extract the crack information. On the other hand, for the above-pavement sparse components, specifically, the points above the pavement 2 mm are chosen as the road marking candidates, and the shape and width information were further employed to extract the road marking. For the low frequency components ***f***, in order to obtain the slowly changing deformable distresses, such as rutting, on the basis of low frequency components, further processing such as envelope information acquisition and deformation judgment and standard contour extraction [43] can be used to obtain information of pavement deformation distresses. At last, for the vibration component ***t***, since it is difficult to verify the obtained macroscopic texture, we can qualitatively analyze the vibration component only through the pavement with different degrees of texture to reflect the degree of texture fluctuation.

## 4. Experimental Results and Discussion

In this section, we first demonstrate the 3D pavement data and its decomposed components, and then verify the validity of the compositions using the methods shown in Figure 7.

For the experimental data, the resolution in elevation direction *R_z* is about 0.2 mm, the resolution in profile direction *R_x* is about 1 mm, and the resolution in driving direction *R_y* is optional in the range of 1–5 mm.

### 4.1. 3D Pavement Data Decomposition Results

There two variable parameters in above mentioned 3D-PCDM, the cut-off frequency *fc* was analyzed as 0.002 for the low-frequency components allowed in the profile. The total variation de-noising parameters λ was set as 1.25. Here we will illustrate the influence of these two parameters through some instances of actual pavement profiles, and to further verify the suitability of above parameters’ values.

**Figure 8:**
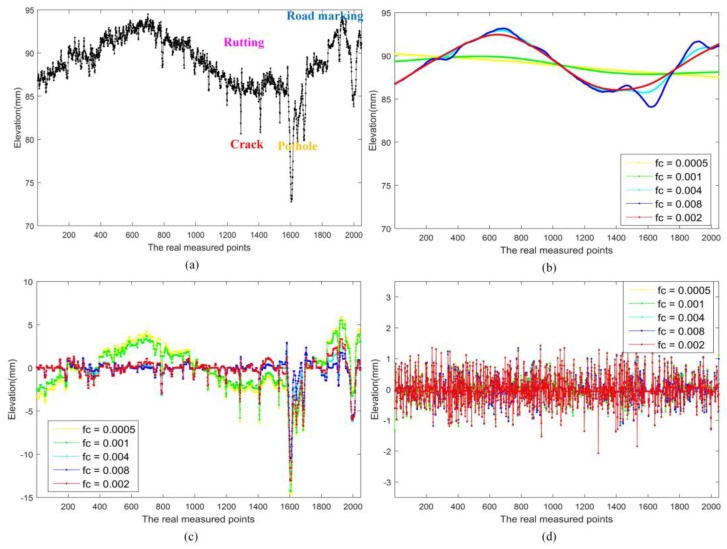
Pavement profile Example 1 and corresponding decomposition with different *fc* and fixed λ, (**a**) profile data; (**b**) low frequency component with different *fc*; (**c**) sparse component with different *fc*; and (**d**) vibration component with different *fc*.

**Figure 9:**
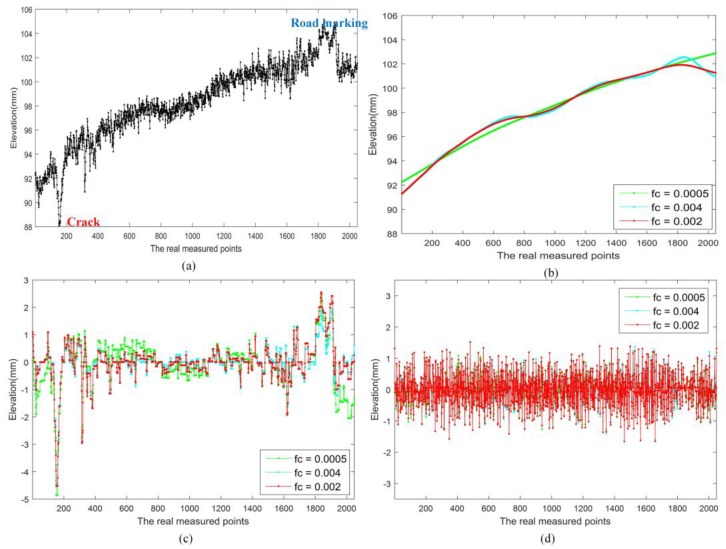
Pavement profile Example 2 and corresponding decomposition with different *fc* and fixed λ, (**a**) profile data; (**b**) low frequency component with different *fc*; (**c**) sparse component with different *fc*; and (**d**) vibration component with different *fc*.

In Figure 8 and Figure 9, there are different types of distresses and performance indicators in the profiles, the cut-off frequency *fc* was varied from 0.0005 to 0.008, and the parameters λ was set as 1.25. It can be found that, if the *fc* smaller than 0.002, the low frequency components could not fit the profiles well, and sparse components also contain some low frequency components. While if the *fc* lager than 0.002, the low frequency could not reflect the depth variation of slow deformation exactly, and there some larger anomalies in the sparse components. These also show that the way we choose the values of parameter *fc* in Section 3.1 is reasonable. In 3D-PCDM, the parameter λ was related to the relative fluctuation of texture in the profile.

**Figure 10:**
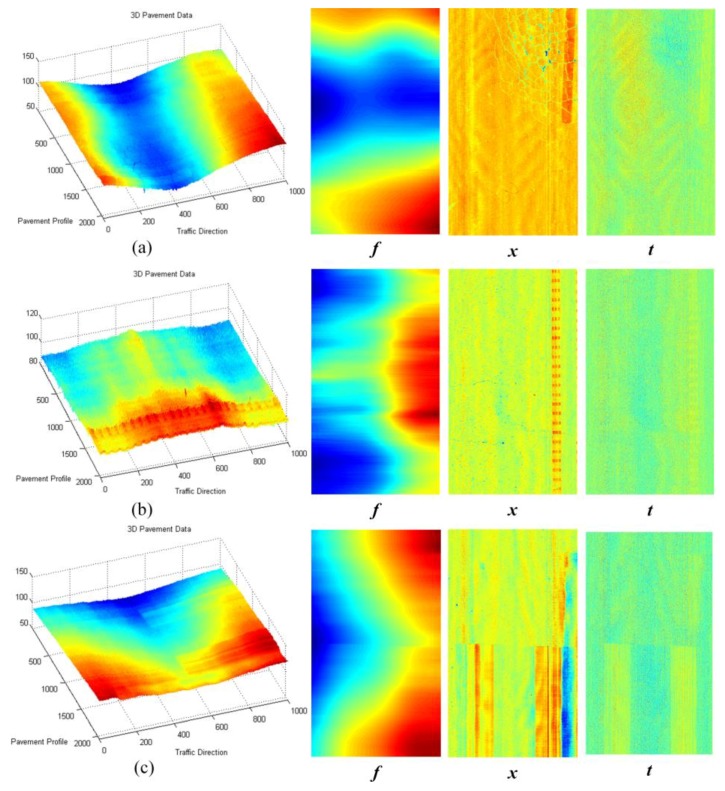
Illustration of 3D pavement data and corresponding decomposition results.

After continuous profile 3D-PCDM processing and corresponding splicing of the same type of components, the corresponding 3D depth maps of different components can be obtained. Figure 10 illustrates more 3D pavement data and the corresponding decomposition results, the leftmost column are original 3D pavement data, and the right ***f***, ***x***, ***t*** represent the low-frequency components, sparse components, and vibration components, correspondingly. It can be seen from Figure 10 that the low-frequency components of the 3D pavement data mainly contain the cross-slope information (due to the vehicle vibrates up and down) [3,13] and certain pavement deformations, while the sparse components contain some crack information, road marking information, and certain pothole information. Moreover, the vibration components reflect the rough texture of the pavement surface, especially in Figure 10c, the difference between the roughness of the surface of the zebra marking and the roughness of the asphalt pavement surface can be reflected. 

The 3D data in Figure 10 are mainly asphalt pavements, including highway pavements and municipal pavements. The original 3D data contain complex pavement information, including vertical vehicle vibration information, pavement distress information, and pavement performance indicators. Typical pavement diseases and indicators can be reflected in the decomposed component data, especially in sparse components and vibration components. From the decomposition results in Figure 10, the proposed 3D-PCDM method can be used as an effective component decomposition method for laser scanning 3D pavement data. 

### 4.2. Evaluation of Components Effectiveness

In order to further verify the effectiveness of the decomposition components, according the validation methods in Figure 7, we conduct some experiments to detect the pavement distresses and performance indicators on different real measured pavement sections. 

**Figure 11:**
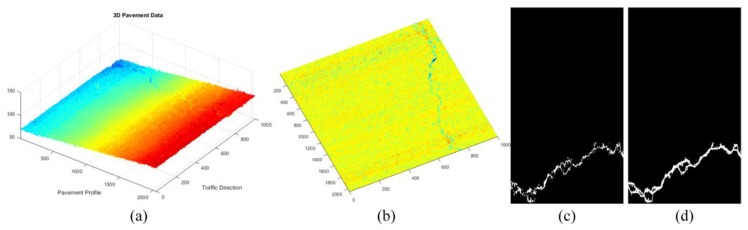
Sparse components and the corresponding pavement traverse cracks information, (**a**) 3D pavement data; (**b**) the corresponding sparse components; (**c**) crack information; (**d**) crack ground truth information.

**Figure 12:**
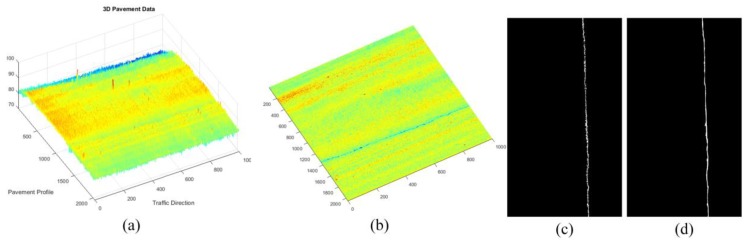
Sparse components and the corresponding pavement longitudinal cracks information, (**a**) 3D pavement data; (**b**) the corresponding sparse components; (**c**) crack information; (**d**) crack ground truth information.

**Figure 13:**
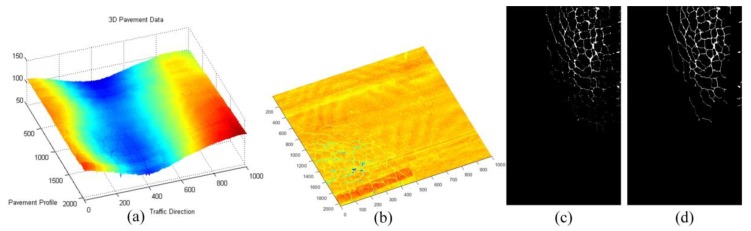
Sparse components and the corresponding pavement alligator cracks information, (**a**) 3D pavement data; (**b**) the corresponding sparse components; (**c**) crack information; (**d**) crack ground truth information.

For the sparse components ***x***, we mainly verify the effectiveness of sparse components through the accuracy of cracks and road marking. The below-pavement sparse components include the cracks candidates and the sharply changing pothole edges information. In Figure 11, Figure 12 and Figure 13, there are cracks in the data, the decomposed sparse components were shown in Figure 11b, Figure 12b and Figure 13b, the selected crack candidates were shown in Figure 11c, Figure 12c and Figure 13c, and the Ground Truth was shown in Figure 11d, Figure 12d and Figure 13d. Then, to quantitatively evaluate the crack extraction results, we employed a classical effective buffered Hausdorff distance metric [3,27,50,51] by comparing the detected cracks with the ground truth cracks, the buffer parameter *L* was heuristically chosen to be 20 for our experiments. The buffered Hausdorff score ranges from 0 to 100, where 100 indicates perfect crack segmentation. For the experimental data in Figure 11, Figure 12 and Figure 13, the accuracy of crack detection is shown in Table 1. It can be seen that all the Buffered Hausdorff Scores are above 92, these show that the sparse components obtained by the decomposition model have higher accuracy.

**Figure 14:**
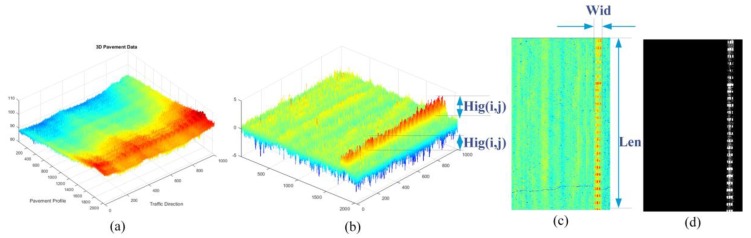
Sparse components and the corresponding road marking information, (**a**) 3D pavement data; (**b**,**c**) the corresponding sparse components and 3D road marking information; (**d**) road marking information.

On the other hand, the above-pavement sparse components, specifically include road markings in the data, the decomposed sparse components were shown in Figure 14b, the selected road marking candidates were shown in Figure 14d, and the method of calculating the 3D information of the marking line as shown in Figure 14b,c.

**Figure 15:**
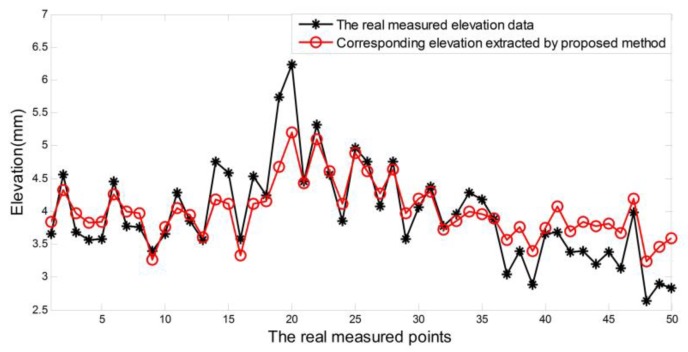
Real measured elevation data and the corresponding marking elevation extracted from the decomposed sparse components.

The road marking information obtained from decomposed sparse components contain not just the location information, but also the accurate length, width, and elevation dimension measurement information of the markings. For the aforementioned experimental data, parts of measurement information—mainly the elevation distribution information—are employed for evaluation. To evaluate the effectiveness and the accuracy of the measurement results, the correlation with real measured data is a main indicator. To get the real measured data, we have selected 50 measuring points on the real pavement section (about 200 m). The real measured elevation data was obtained through a vernier caliper (accuracy: 0.02 mm) and the level ruler to obtain the height of protuberance vibration markings (In order to weaken the effect of the pavement texture depth, we mainly measure the protruding part of the markings). The real measured elevation data and the corresponding marking elevation extracted by the proposed method are illustrated in Figure 15. The correlation of these two data is about 92.87%. It can be observed that the marking elevation information obtained from decomposed sparse components is more stable than the real measured data, and the differences between these two data are mostly within 1 mm. This is mainly due to the unavoidable impact of pavement texture depth on real measured points, although we try to take the pavement surface texture center as possible when we choose the datum surface. In fact, the 3D road markings’ information obtained from sparse components is more comprehensive, and can even be used to evaluate the wear information of the road markings.

**Figure 16:**
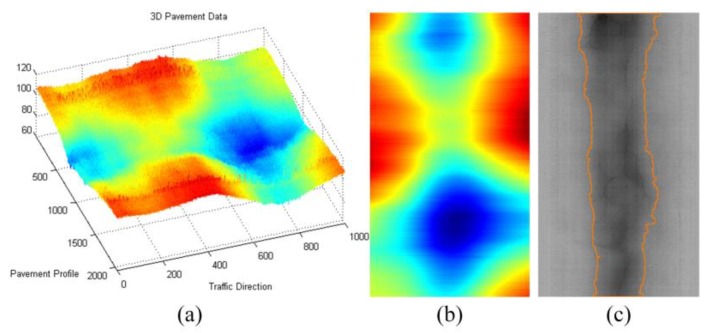
The low-frequency components and the corresponding pavement distresses information, (**a**) 3D pavement data; (**b**) corresponding low-frequency components; (**c**) rutting information.

For the low frequency component ***f***, in order to further obtain the slowly changing deformable distresses—such as rutting based on low frequency components—further processing including envelope information acquisition and deformation judgment and standard contour extraction [43] can be used to obtain information of pavement deformation distresses. Figure 16 illustrates the decomposed low frequency component and further extracted rutting information by the method mentioned in [43]. 

**Figure 17:**
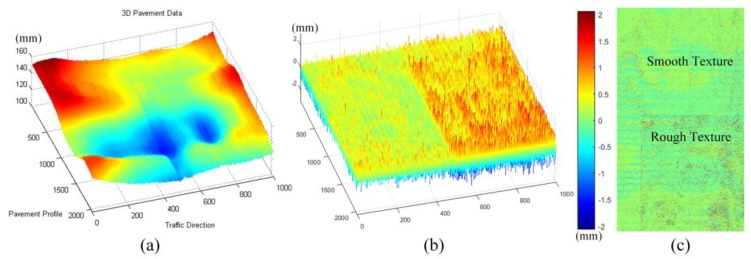
Vibration components and the corresponding pavement texture information, (**a**) 3D pavement data; (**b**) corresponding vibration component; (**c**) rough and smooth texture information.

At last, for the vibration component ***t***, since it is difficult to verify the obtained macroscopic texture, we can qualitatively analyze the vibration component only through the pavement with different degrees of texture to reflect the degree of texture fluctuation. Figure 17 illustrates the decomposed vibration component, it can be seen from the figure that the obtained vibration components can reflect the rough and smooth texture information in pavement surface, and the texture depth information can be further analyzed from obtained vibration components.

As can be seen from the Figure 11, Figure 12, Figure 13, Figure 14, Figure 15, Figure 16 and Figure 17, the three kinds of decomposed components are all associated with the actual pavement distresses and performance indicators. The accuracy of the decomposed components can be verified, the effectiveness and accuracy of the sparse components ***x*** are verified by actual cracks and road markings, the accuracy of extracted sparse components is over 92.75%. Typically, the texture of the pavement is generally 1–2 mm. How to further obtain the texture depth of the pavement to obtain more accurate elevation data is one of our follow-up works.

### 4.3. Discussion

**Figure 18:**
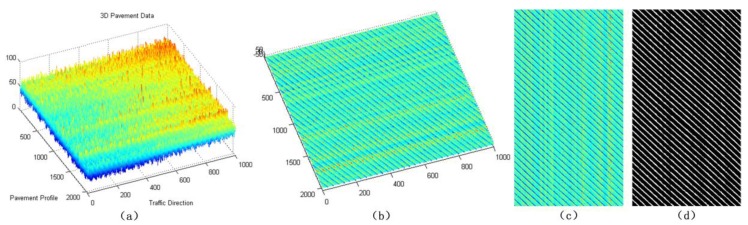
The 3D airport asphalt pavement Data 1 and corresponding sparse components, (**a**) 3D data from airport asphalt pavement; (**b**,**c**) corresponding sparse components; (**d**) pavement groove information extracted from sparse components.

**Figure 19:**
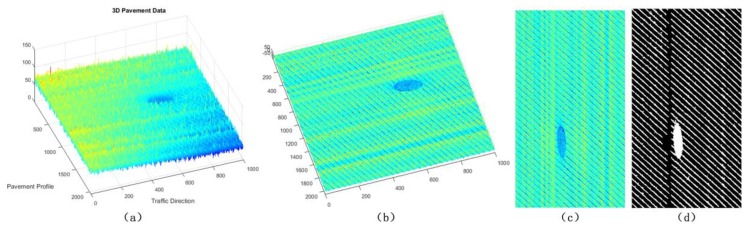
The 3D airport asphalt pavement Data 2 (with pothole) and corresponding sparse components, (**a**) 3D data from airport asphalt pavement; (**b**,**c**) the corresponding sparse components; (**d**) pavement groove and pothole information extracted from sparse components.

It is important to note that the proposed decomposition model can be used as a basis for many typical 3D pavement distresses and performance indicators extraction methods, not only the processing basis of the method shown in Figure 7, and it can reduce the impact of other indicators on the interested indicator. For the experimental analysis in Section 4.1 and Section 4.2, the proposed 3D-PCDM mainly validated through some common types of pavement distresses and performance indicators in the asphalt highway pavement and asphalt municipal pavement. To further verify the applicability and effectiveness of the proposed 3D-PCDM method to other performance indicators in other types of pavements. Some 3D airport asphalt pavement data which contain grooves [13,24] have been employed, as illustrated in Figure 18 and Figure 19. Theoretically, the elevation information of the grooves belongs to sparse components in the pavement profiles. From Figure 18 and Figure 19, it can be seen that the groove features in the original 3D data (Figure 18a and Figure 19a) are not obvious enough. After adopting the proposed decomposition model, the elevation feature of the grooves in the sparse component is obviously reflected, and the groove information obtained by the sparse component is very complete in Figure 18d and Figure 19d. This also shows that the proposed 3D laser scanning pavement components decomposition model can be used as a basis for the extraction of many typical 3D pavement distresses and performance indicators. In our follow-up work, we will further verify the application of this method to more indicators in more different pavements, such as cement pavement, needs further verification.

Pavement texture information obtained from traditional systems are restricted on either a small portion on pavement surface or one line-of-sight profile, and the currently used texture indicators, such as mean profile depth (MPD) and mean texture depth (MTD), only reveal partial aspects of texture property [52,53]. With the emergence 3D laser scanning technology, acquiring full-lane 3D pavement data at sub-millimeter resolution and at highway speeds has been made possible. Thus, new types of texture indicators need to be calculated to represent various texture properties for pavement friction estimation. It can be seen from Figure 10c and Figure 17 that the obtained vibration component can reflect the difference in the pavement macro texture [52,53]. Thus, the vibration components obtained by 3D-PCDM are beneficial to the quantitative analysis of the pavement macro texture. Although this paper does not give a quantitative macro texture analysis based on this component, it is mainly due to the problem that the current texture verification problem has not been solved. On the other hand, pavement texture information is always related to pavement distresses, and it is also an important index for pavement maintenance and management. Thus, the evaluation of the 3D texture an important part of subsequent research on 3D pavement data analysis.

All abovementioned experiments are verified by 3D asphalt pavement data (highway pavement, municipal pavement, and airport asphalt pavement), but whether the algorithm can be applied to other structures such as cement pavements and concrete structures [54] needs further analysis and verification. In the analysis of the 3D cement pavement data, the characteristics of low frequency components and vibration components need further analysis and modelling. The parameters involved in proposed 3D-PCDM should be modified for the cement pavement. This is another limitation and deficiency of this method, that is, the method may need further adjustment for cement pavement.

## 5. Conclusions

In view of the rich and complex pavement information contained in the high-precision 3D laser scanning pavement data and the requirement for the simultaneous acquisition of various pavement distresses and performance indicators in practical application, this paper proposes a 3D pavement components decomposition model (3D-PCDM) which decomposes the 3D pavement profiles into sparse component ***x***, low-frequency component ***f*,** and vibration component ***t***. 3D-PCDM provides a basis for simultaneously analyzing various pavement distresses and performance indicators for 3D laser scanning pavement data. By analyzing the frequency characteristics and sparse characteristics of pavement distresses and performance indicators—including the cracks, road markings, rutting, potholes, textures—the cut-off frequency *fc* of low frequency components and high frequency components in 3D pavement profile data was first obtained from the perspective of 3D pavement profiles. Then, a designed high-pass filter was employed to separate low-frequency component ***f***. Furthermore, sparse component ***x*** and the vibration component ***t*** are separated by total variation de-noising which based on sparse characteristics. The ***x*** can be used to characterize the location and depth information of sparse and sparse derived signals such as cracks, road marks, pothole edges, and grooves in profiles. The ***f*** can be used to determine the slow deformation of pavement or the acquisition of standard contour. While the ***t*** reflects the fluctuation of the pavement material particles. Experimental tests were conducted using actual pavement 3D data, the decomposed components can be obtained by 3D-PCDM. The effectiveness and accuracy of the sparse components are verified by actual cracks and road markings, the accuracy of extracted sparse components is over 92.75%, and the effectiveness of the vibration components is verified by pavement texture. 

The quantitative analysis of pavement macro texture based on 3D-PCDM vibration component need further studied. Moreover, the relationship between macro texture information and other pavement distresses also needs further research. On the other hand, whether the algorithm can be applied to other structures such as cement pavements and concrete structures needs further analysis and verification. In addition, the current proposed method only explores the information in the transverse profile direction. In the future, the information about driving direction in 3D pavement data will be used for the analysis of pavement distresses and performance indicators information.

## Figures and Tables

**Table 1:** Buffered Hausdorff scores of test data in Figure 11, Figure 12 and Figure 13.

Crack Types	Traverse Cracks	longitudinal Cracks	Alligator Cracks	Average Score
Buffered Hausdorff Score	92.75	95.89	94.13	94.25
